# Ionizing radiations sustain glioblastoma cell dedifferentiation to a stem-like phenotype through survivin: possible involvement in radioresistance

**DOI:** 10.1038/cddis.2014.509

**Published:** 2014-11-27

**Authors:** P Dahan, J Martinez Gala, C Delmas, S Monferran, L Malric, D Zentkowski, V Lubrano, C Toulas, E Cohen-Jonathan Moyal, A Lemarie

**Affiliations:** 1 INSERM UMR 1037, Centre de Recherches en Cancérologie de Toulouse (CRCT), Université Toulouse III Paul Sabatier, Toulouse, France; 2 Laboratoire d'Oncogénétique, Institut Universitaire du Cancer Toulouse-Oncopole, Toulouse, France; 3 Faculté des Sciences Pharmaceutiques, Université Toulouse III Paul Sabatier, Toulouse, France; 4 INSERM UMR 825, Université Toulouse III Paul Sabatier, Toulouse, France; 5 Service de Neurochirurgie, Centre Hospitalier Universitaire de Rangueil, Université Toulouse III Paul Sabatier, Toulouse, France; 6 Département de Radiothérapie et Oncologie, Institut Universitaire du Cancer Toulouse-Oncopole, Toulouse, France

## Abstract

Glioblastomas (GBM) are some bad prognosis brain tumors despite a conventional treatment associating surgical resection and subsequent radio-chemotherapy. Among these heterogeneous tumors, a subpopulation of chemo- and radioresistant GBM stem-like cells appears to be involved in the systematic GBM recurrence. Moreover, recent studies showed that differentiated tumor cells may have the ability to dedifferentiate and acquire a stem-like phenotype, a phenomenon also called plasticity, in response to microenvironment stresses such as hypoxia. We hypothesized that GBM cells could be subjected to a similar dedifferentiation process after ionizing radiations (IRs), then supporting the GBM rapid recurrence after radiotherapy. In the present study we demonstrated that subtoxic IR exposure of differentiated GBM cells isolated from patient resections potentiated the long-term reacquisition of stem-associated properties such as the ability to generate primary and secondary neurospheres, the expression of stemness markers and an increased tumorigenicity. We also identified during this process an upregulation of the anti-apoptotic protein survivin and we showed that its specific downregulation led to the blockade of the IR-induced plasticity. Altogether, these results demonstrated that irradiation could regulate GBM cell dedifferentiation via a survivin-dependent pathway. Targeting the mechanisms associated with IR-induced plasticity will likely contribute to the development of some innovating pharmacological strategies for an improved radiosensitization of these aggressive brain cancers.

Radiotherapy is, following surgical resection and associated with Temozolomide, the gold standard treatment for glioblastoma (GBM). However, even after the association of surgery and combined chemo/radiotherapy, these invasive and resistant tumors almost systematically recur, with a median overall survival of 14 months.^1^ It is now established that GBM are some very heterogeneous tumors similar to most of the solid cancers.^2^ Recent studies highlighted the presence of a subpopulation of self-renewing and pluripotent GBM stem-like cells (GSCs), also called GBM-initiating cells, among the tumor. These GSC are characterized by their ability to self-renew *in vitro* (neurospheres (NS) formation) and *in vivo*, their higher expression of neural stem cell (NSC) markers (i.e., Olig2, Nestin or A2B5) and stem cell transcription factors (SCTF, i.e., Sox2, Nanog, Gli1 or Oct4), their pluripotent aptitude to differentiate into neurons, astrocytes or oligodendrocytes and their high tumorigenic potential *in vivo* in mice.^3, 4^ In addition, the presence of these GSC may explain the high GBM recurrence rate, as they were shown to be extremely tumorigenic and radioresistant.^3, 5, 6^

Several radioresistance mechanisms have been identified in these GSC. Most of them are in favor of a clonal selection process through the GSC intrinsic resistance to ionizing radiation (IR)-induced cell death,^7, 8^ supported by a better efficiency of DNA-damage repair systems,^6, 9, 10^ a higher level of anti-apoptotic^11, 12^ or pro-survival factors^13, 14, 15^ and a sustained expression of pluripotency maintenance factors such as Notch1,^16^ TGF*β*,^17, 18^ Sonic hedgehog (SonicHH),^19^ STAT3^20^ or Wnt.^21^ Besides this, the influence of the microenvironment could also participate in radioresistance,^17, 22^ as hypoxia, which is a well-known factor of radioresistance,^23, 24^ acidic extracellular pH^25^ and nitric oxide^26, 27^ were shown to be involved in GSC stemness preservation.

However, several studies have put forward the hypothesis that GBM-differentiated cells may be able to dedifferentiate toward a stem-like state when submitted to appropriate stimuli^7^ and then contribute to increase the tumor stem cell pool. This assumption was supported by studies showing that hypoxic conditions, hepatocyte growth factor or Temozolomide could induce such a phenomenon in GBM cells.^23, 28, 29^ Of note, hypoxia was previously shown to induce a similar reprogramming in breast cancer^30^ and neuroblastoma cells,^31^ and our group showed that IR can stabilize HIF1*α* (hypoxia-inducible factor 1*α*) and activate the associated hypoxic pathways in GBM.^32, 33^ This dedifferentiation process was also demonstrated *in vivo* in murine neurons and astrocytes through the expression of GBM-associated oncogenes.^34^ In line with this, recent works showed that IRs were able to induce at short term the expression of stem markers (such as Sox2, Nestin and CD133) in GBM,^35^ without studying the presence of a potential dedifferentiation process. In consequence, we hypothesized that plasticity may occur after radiotherapy in resistant remaining GBM cells. The present study was designed to analyze the long-term effects of radiotherapy on the phenotypic and molecular status of GBM cells isolated from several patient resections and to find out whether or not these cells can dedifferentiate toward a stem-like phenotype in response to IR.

Our present data show in human primary GBM patient cell lines that a subtoxic IR dose can induce at long term the overexpression of a large panel of stem markers in GBM cells, a potentiation of their NS-forming capacity and an exacerbated tumorigenesis in nude mice, indicating an IR-induced dedifferentiation process. We have also identified the inhibitor of apoptosis protein (IAP) survivin as an important regulator of this IR-induced plasticity. In conclusion, we showed here for the first time that radiotherapy is able to sustain a phenotype shift toward stemness in GBM, which may participate in the expansion of the cancer stem-like compartment in GBM after treatment and finally favor a fast recurrence of these aggressive and invasive brain cancers.

## Results

### Characterization of the human primary GBM cells subjected to the IR-induced dedifferentiation protocol

**Figure 1:**
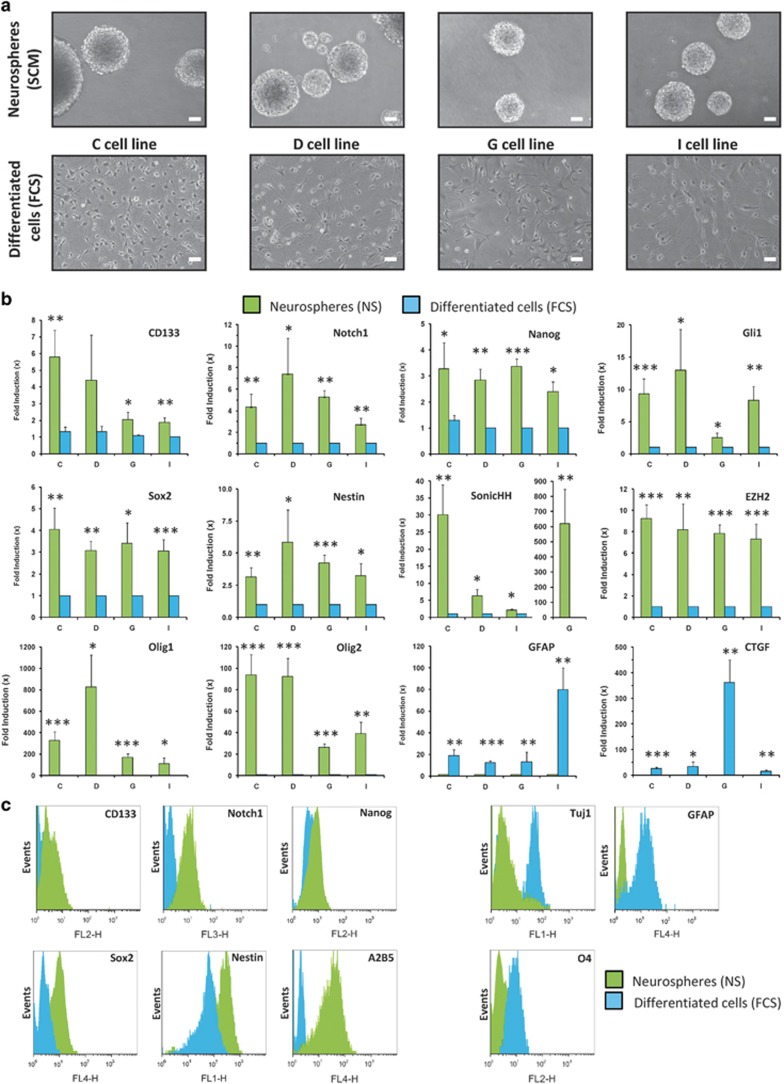
Characterization of the stem and differentiated phenotypes in GSC-enriched NS and FCS-differentiated GBM cultures. (**a**–**c**) GSC-enriched NS cell lines isolated from four patient tumors (C, D, G and I) were kept in SCM medium or allowed to differentiate as adherent GBM cells for at least 15 days in FCS medium. (**a**) Phase-contrast photomicrographs of NS or GBM-differentiated cells. Original magnification: × 10, scale bar: 6 *μ*m. (**b**) Real-time quantitative PCR analysis of the stem (CD133, Notch1, Nanog, Gli1, Sox2, Nestin, SonicHH, EZH2, Olig1 and Olig2) and differentiation (GFAP and CTGF) markers in NS or GBM-differentiated cells for the C, D, G and I cell lines. Shown are the fold inductions expressed as means±S.E.M. of at least three independent experiments. ^∗^*P*<0.05, ^∗∗^*P*<0.01, ^∗∗∗^*P*<0.001 compared with the related control. (**c**) Immunofluorescence FACS analysis of stem (CD133, Notch1, Nanog, Sox2, Nestin and A2B5) and differentiation (GFAP, Tuj1 and O4) markers in NS or GBM-differentiated cells. The results depicted were representative of three independent experiments (G cell line) and were reproduced in all the cell lines

**Figure 2:**
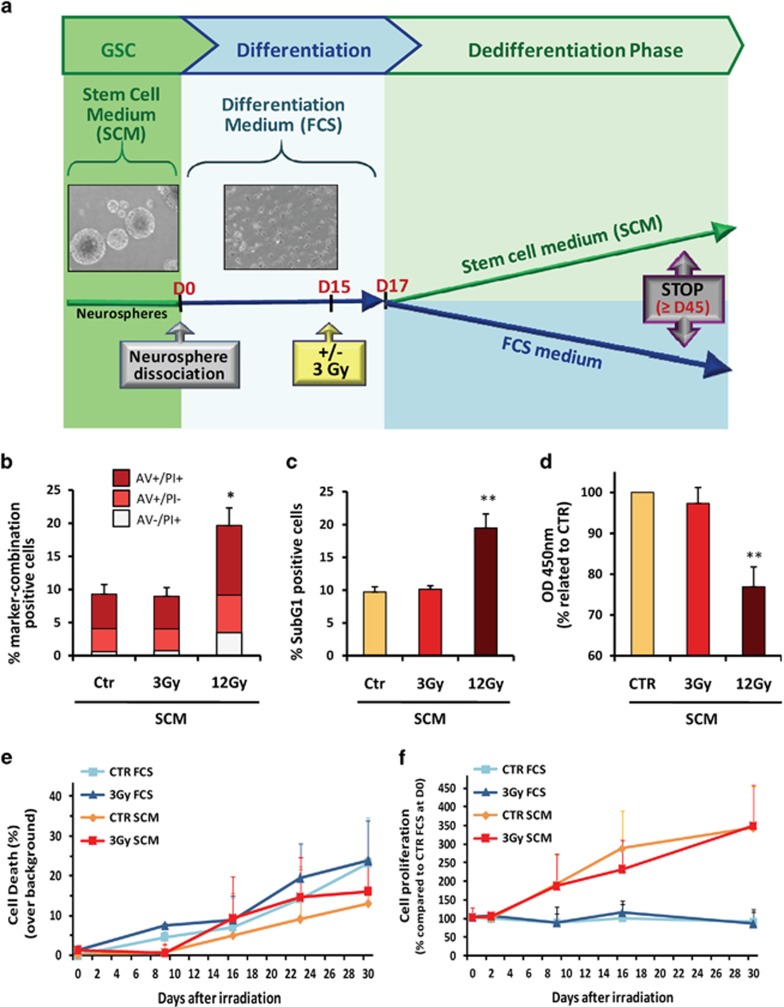
Overview of the long-term dedifferentiation protocol and assessment of the irradiation dose. (**a**) As described in the Materials and Methods section, GSC-enriched NS were isolated from patient samples and cultured in a specific SCM medium. NS cells were then dissociated and placed into a differentiating medium with FCS (FCS medium) for at least 15 days, to allow an optimum differentiation. Adherent differentiated GBM cells were then subjected or not to a 3-Gy irradiation and were placed 2 days after in fresh FCS medium, to keep them fully differentiated, or in SCM medium to favor a possible dedifferentiation process. The totality of the cells was finally collected at the end of the protocol, which coincided to the apparition of NS in the culture supernatant, in order to be analyzed. (**b**–**f**) Absence of effects of a 3-Gy irradiation on viability and proliferation of GBM cells during the dedifferentiation protocol. Differentiated GBM cells were subjected or not (Control, CTR) to a 3-Gy irradiation and placed 2 days after in SCM medium for 5 additional days, according to the dedifferentiation protocol (**b**–**d**) or for different time points during this dedifferentiation protocol (**e** and **f**) The dose of 12 Gy was chosen as a positive control for cell death induction. At 7 days post IR, cells were analyzed either by FACS for AV/PI double staining of both apoptotic and necrotic cells (**b**) and PI staining of the Sub-G1 population (**c**), or through WST-1 staining using a spectrophotometric determination of the dye absorbance at 450 nm for the quantification of cell viability and proliferation (**d**). For kinetic studies, cell death was assessed by FACS, by AV/PI staining and expressed as percentages over background (**e**), and cell count was performed to estimate the cell proliferation rate in the indicated culture condition (**f**). Shown are the means±S.E.M. of at least three independent experiments. ^∗^*P*<0.05, ^∗∗^*P*<0.01 compared with the related control

To study the hypothesis of an IR-induced plasticity, four GSC cell lines (C, D, G and I) previously established in our group from patient surgical GBM samples and cultured as GSC-enriched NS^29^ were forced to differentiate in fetal calf serum (FCS) medium for at least 15 days, leading to a dramatic change in their cellular morphology and adhesion properties, and to the loss of their ability to generate NS by self-renewal (Figure 1a). These differentiated GBM cells were then subjected or not to a 3-Gy irradiation and placed 2 days after in either FCS or stem cell medium (SCM), in order to maintain a differentiated status or to favor a possible reversion to a stem phenotype, respectively (Figure 2a). These culture conditions were maintained until the generation of NS in the medium, testifying of the reappearance of the *in vitro* self-renewal ability. The long-term effects of irradiation were then analyzed at a molecular level by RT-qPCR, western blotting or FACS analysis, and *in vivo* by the tumorigenicity of the treated cells in orthotopically xenografted nude mice.

In order to fully characterize the differentiated GBM cells subjected to this dedifferentiation protocol, we first checked by RT-qPCR the expression of several stem and differentiation markers in NS-derived cells and their differentiated counterparts. We observed that in all NS cell lines, the expression of the largely described stem markers CD133, Notch1, Nanog, Gli1, Sox2, Nestin, SonicHH, EZH2 and Olig1/Olig2,^3, 36, 37^ was markedly and significantly repressed after a 15-day forced differentiation (Figure 1b). On the contrary, the expression of several differentiation markers such as GFAP (glial fibrillary acidic protein) or connective tissue growth factor (CTGF)^3, 38^ was potently upregulated in FCS-differentiated cells but almost undetectable in NS (Figure 1b). We confirmed by FACS that the cell surface stem markers CD133, Notch1 and A2B5^4^ were totally abrogated in the differentiated cells, as well the SCTF Nanog and Sox2 (Figure 1c). The NSC protein Nestin, highly expressed in glioma and particularly in GSC,^39, 40^ also appeared markedly decreased. Finally, we observed the appearance in these differentiated cells of the three lineage-specific differentiation markers GFAP, O4 and TUJ1 (*β*3-Tubulin),^29^ pointing the ability of this differentiation process to exploit the GSC pluripotency to generate, respectively, tumor astrocytes, oligodendrocytes and neurons (Figure 1c). Of note, these differentiation markers were not expressed in the NS cells. The results were reproduced in all cell lines (data not shown).

### Absence of alteration of both cell viability and proliferation in response to a 3-Gy irradiation

As previously mentioned, it appeared necessary in this study to avoid any clonal selection after irradiation in order to only study the potential dedifferentiation process induced by IR. As a consequence, our protocol required a non-toxic IR dose. In that way, we performed some clonogenic NS formation assays at different IR doses (0–10 Gy) and we observed that no significant toxicity can be detected at 3 Gy or lower (data not shown). Next, we checked whether or not this 3-Gy subtoxic dose was able to alter cellular viability or proliferation during the dedifferentiation process. We also used a 12-Gy dose as a positive control for toxicity. Using annexin V (AV)/propidium iodide (PI) double staining and SubG1 detection to assess necrosis and apoptosis after a 3-Gy irradiation, we failed to observe any viability impairment 7 days post IR (Figures 2b and c) or along the whole dedifferentiation process (Figure 2e). Similarly, we did not see, using WST1 assay, any alteration in cell proliferation at 7 days post IR (Figure 2d). A similar statement was made during the whole dedifferentiation protocol, as we could not observe any change in the cell count between the control and the 3-Gy-irradiated groups (Figure 2f). As expected, the 12-Gy dose induced a decrease in cell proliferation and an increase of apoptotic and necrotic cell death as soon as 7 days post IR (Figures 2b, c and d).

### Potentiation of the long-term acquisition of a stem-like phenotype by IR in GBM differentiated cells

**Figure 3:**
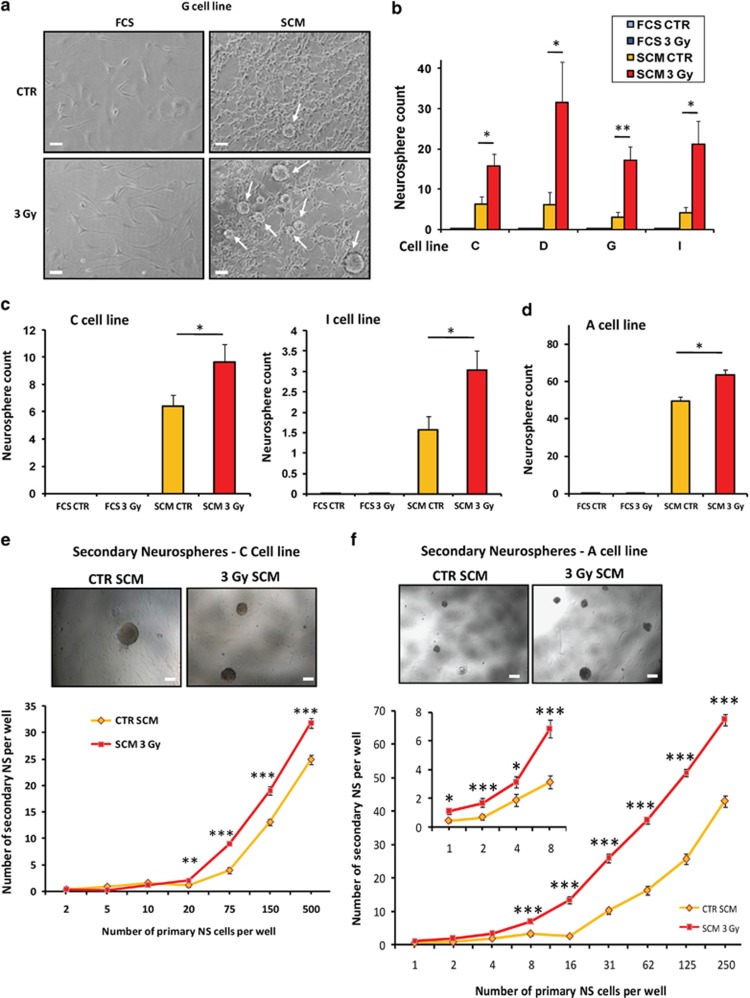
Increased ability to generate NS in GBM-differentiated cells subjected to a 3-Gy subtoxic irradiation. Cells treated or not by a 3-Gy irradiation and placed 2 days after in either FCS or SCM medium for long-term culture were analyzed in order to evaluate the number of NS generated in the culture supernatant. (**a**) Phase-contrast photomicrographs of GBM cells at the end of the dedifferentiation protocol. Arrows indicate the presence of NS. Original magnification: × 10, scale bar: 6 *μ*m. (**b–d**) Quantification of the number of generated NS at the end of the dedifferentiation protocol for the four cell lines C, D, G and I in irradiated (3 Gy) or untreated (CTR) GBM cells kept in SCM medium. (**b**) NS were counted and results are expressed per 25-cm^2^ flasks. Results are expressed as the means±S.E.M. of at least three independent experiments. ^∗^*P*<0.05, ^∗∗^*P*<0.01 compared with the related control. (**c**) NS were also generated from differentiated cells through the use of a dilution assay at low density and were counted at different dilutions in each well of a 24-well plate. The results are shown for the C and I cell lines (20 000 cells/well). ^∗^*P*<0.05. (**d**) Before the NS generation assay, A2B5-negative differentiated cells were sorted as described in the Materials and Methods section (A cell line). The generated primary NS were then counted at the end of the dedifferentiation protocol. Results are expressed as the means±S.E.M. of three independent experiments. ^∗^*P*<0.05 (**e** and **f**) Primary NS were generated from differentiated cells with (**f**) or without (**e**) an optional FACS sorting of the A2B5-negative differentiated cells. These primary NS were subsequently dissociated and plated in 96-well plates at different low cell densities, to study their ability to generate secondary NS through limiting dilution assays. ^∗^*P*<0.05. ^∗∗^*P*<0.01, ^∗∗∗^*P*<0.001 compared with the related CTR SCM condition. Representative phase-contrast photomicrographs were shown for each conditions (original magnification: × 4, scale bar: 17 *μ*m)

Using these characterized GBM differentiated cells irradiated by a 3-Gy subtoxic dose, we subjected or not the cells to a medium change and place them either in FCS medium to preserve their differentiated status or in SCM medium to favor the potential appearance of a stem phenotype in a permissive environment (Figure 2a). NS did not appear in cells kept in FCS medium, even after IR (Figure 3a). However, we observed after at least 4 weeks of culture post IR (25 cm^2^ culture flasks) the generation of NS in SCM medium (Figure 3a). This was noticed at a basal level in non-irradiated control cells (CTR) and was greatly potentiated by IR by two- to six fold according the cell line (Figures 3a and b). We also plated GBM-differentiated cells at low densities in 24-well plates in order to perform a NS generation assay adapted from the classic limiting dilution assay used generally for GSC.^41^ Using this protocol, we were able to confirm that FCS-maintained cells, with or without IR, failed to generate NS. On the contrary, SCM-cultured cells gave rise to NS at long term and this generation was greatly potentiated after irradiation (Figure 3c). We also performed this NS generation assay after sorting the differentiated GBM cells (negative for A2B5; Tchoghandjian *et al.*^4^) by FACS analysis in order to discard some eventual remaining GSC in our differentiated cell cultures and we observed the same increase in NS generation in response to irradiation (Figure 3d). Finally, we also established through the use of a classic limiting dilution assay^41^ that these primary NS generated in SCM medium were able to give rise to secondary NS, with a marked increased ability for primary NS-derived cells obtained after a 3-Gy irradiation. This was observed whether the differentiated cells were sorted or not before irradiation (Figures 3e and f).

**Figure 4:**
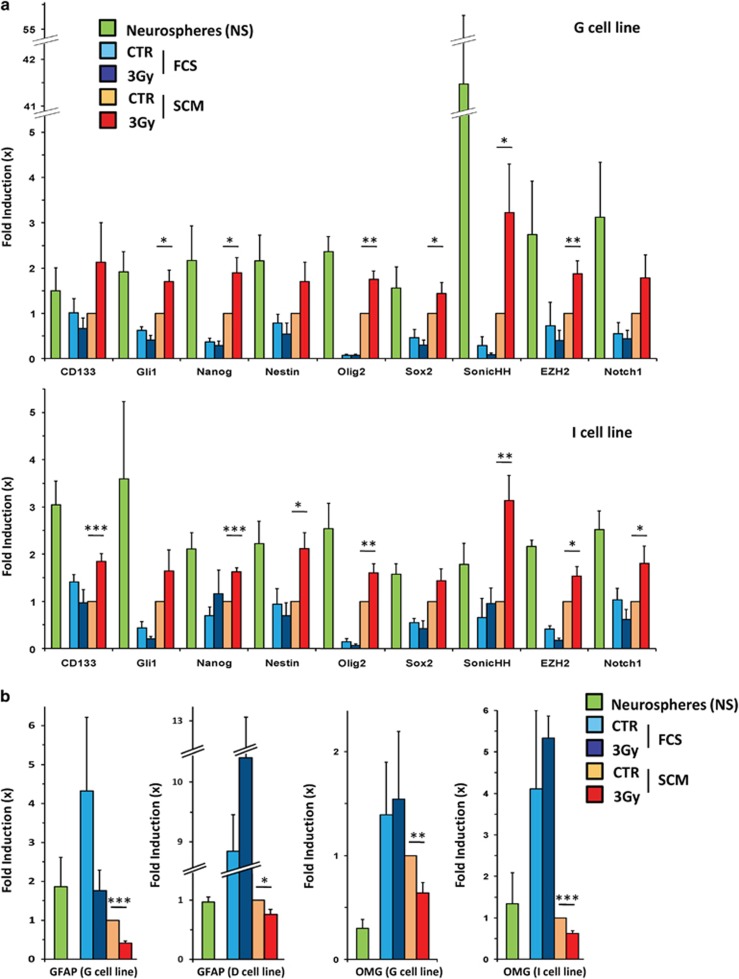
Overexpression of stemness markers and downregulation of differentiation markers at the RNA level in GBM cells after a 3-Gy irradiation. Differentiated GBM cells treated or not by a 3-Gy irradiation and placed 2 days after in either FCS or SCM medium for long-term culture were analyzed by real-time quantitative PCR (see Materials and Methods) at the end of the dedifferentiation protocol. RNA expression level of the indicated stem (**a**) or differentiation markers (**b**) in GBM-differentiated cells subjected to the dedifferentiation process for the indicated cell lines. The RNA expression levels of these different markers were also shown for NS cells as a control, as these NS are enriched in GSC. Shown are the fold inductions relative to the CTR SCM condition expressed as means±S.E.M. of at least three independent experiments. ^∗^*P*<0.05, ^∗∗^*P*<0.01, ^∗∗∗^*P*<0.001 compared with the related CTR SCM condition

We then analyzed the expression of a large panel of stem markers at the end of the dedifferentiation protocol. We demonstrated by RT-qPCR that the mRNA expression levels of several well-established stem markers, such as Nanog, Olig2, SonicHH and EZH2, were markedly increased in 3-Gy-treated cells grown in SCM medium compared with untreated cells kept in the same medium (Figure 4a). We did not observe this IR-induced potentiation in FCS condition. Similar results were obtained for all the cell lines (data not shown). We analyzed in parallel the expression of several differentiation markers and showed that the astrocytic marker GFAP and the oligodendrocytic marker oligodendrocyte-myelin glycoprotein^42^ were accordingly downregulated in SCM medium and that this decrease was amplified after irradiation (Figure 4b).

**Figure 5:**
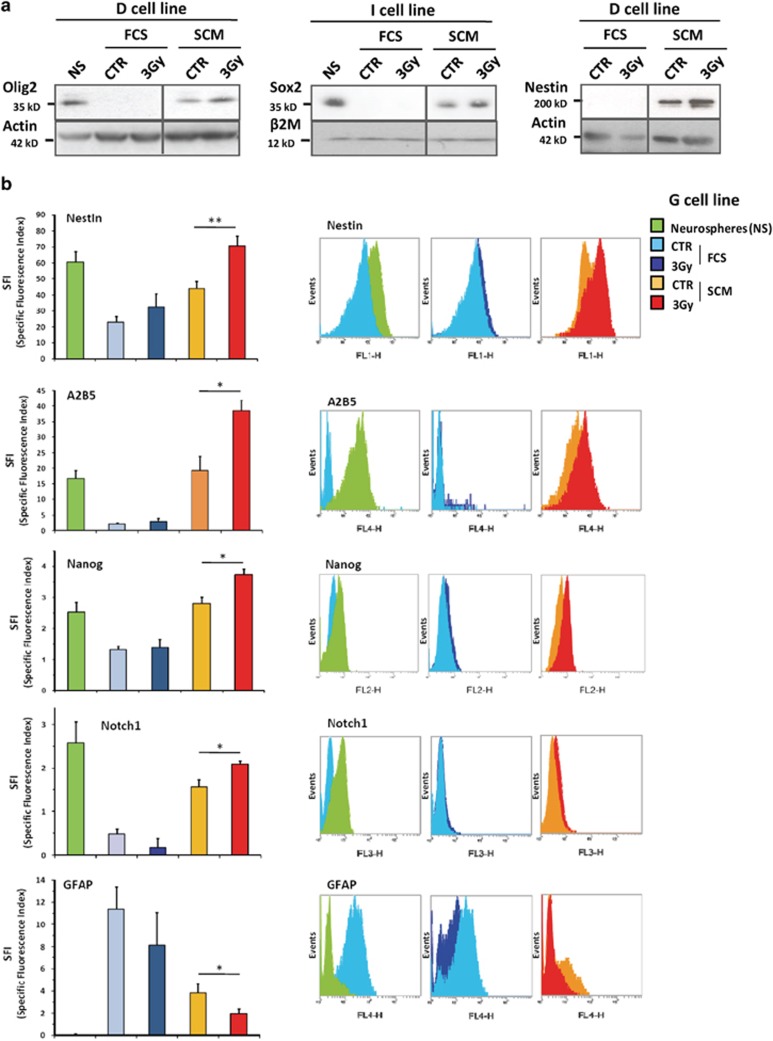
Overexpression of stemness markers and downregulation of differentiation marker at the protein level in GBM cells after a 3-Gy irradiation. Differentiated GBM cells treated or not by a 3-Gy irradiation and placed 2 days after in either FCS or SCM medium for long-term culture were analyzed either by western blotting (**a**) or FACS immunofluorescence (**b**) at the end of the dedifferentiation protocol. Protein expression levels in NS cells were shown as a control for the stem condition. (**a**) Western blotting analysis of the stem markers Olig2, Sox2 and Nestin. Equal gel loading and transfer efficiency were checked with anti-actin or *β*2-microglobulin (*β*2M) antibodies. Blots were representative of at least three independent experiments in the indicated cell line and were reproduced in all the cell lines. (**b**) Immunofluorescence analysis performed by FACS of the stem (Nestin, A2B5, Nanog and Notch1) and differentiation (GFAP) markers in the G cell line. The SFI allowed to evaluate the marker expression level (see Materials and Methods). Results are expressed as the means±S.E.M. of at least three independent experiments in the G cell line and were reproduced in all the cell lines. ^∗^*P*<0.05, ^∗∗^*P*<0.01 compared with the related CTR SCM condition. For each marker, some representative FACS plot overlays (NS *versus* CTR FCS, CTR FCS *versus* 3 Gy FCS and CTR SCM *versus* 3 Gy SCM) were depicted

To confirm these data at the protein level, we demonstrated by western blotting in all the cell lines that long-term culture in SCM medium of irradiated differentiated GBM cells greatly enhanced the expression of Olig2, Sox2 and Nestin compared with untreated cells kept in SCM medium, which nevertheless showed a slight increase of these stem markers compared with FCS-differentiated cells (Figure 5a), as observed at the RNA level (Figure 4a). However, short-term analyses (2–7 days post IR) demonstrated that this stem markers overexpression can exclusively be observed after IR in SCM medium, without any effect in FCS-cultured cells or in SCM-control cells, pointing out the predominant role of irradiation for stem markers induction (Supplementary Figure 1a). In order to measure more accurately the overexpression of different stem (Nestin, A2B5, Nanog and Notch1) and differentiation (GFAP) markers, we demonstrated by FACS analysis in all the cell lines that 3-Gy-irradiated cells cultured in SCM medium either markedly upregulated these stem markers or downregulated GFAP compared with the control SCM condition (Figure 5b). In addition, we were able to show that this phenomenon was generalized to the whole cell population and did not seem to be due to the expansion of some particular cell clones within the cellular population.

### Increased tumorigenic potential *in vivo* in IR-dedifferentiated GBM cells

**Figure 6:**
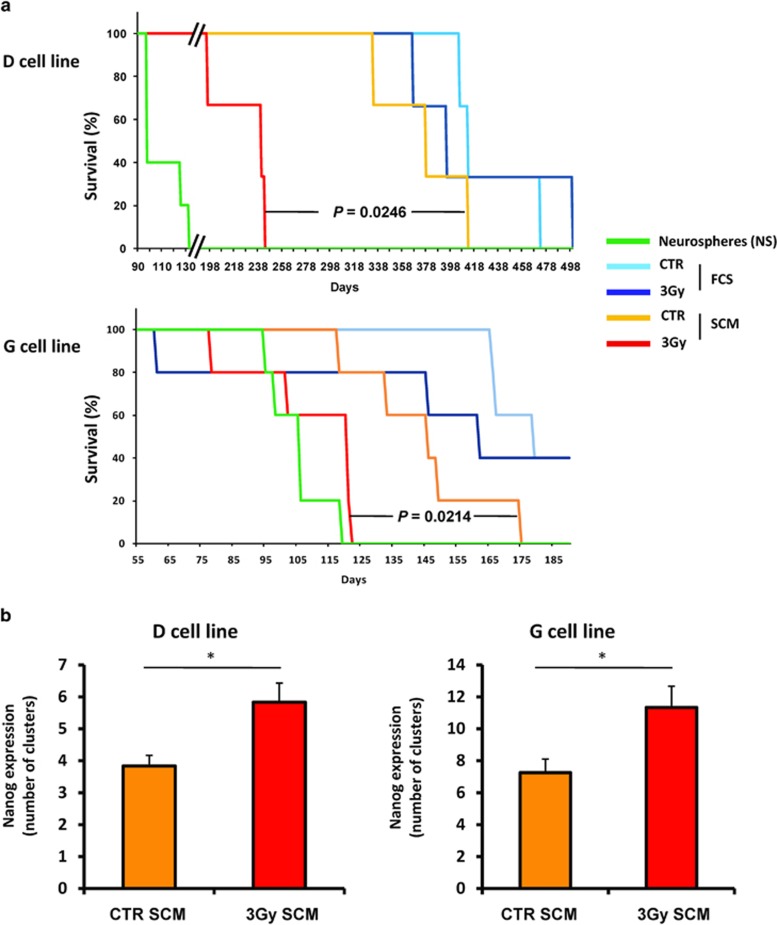
Increased *in vivo* tumorigenicity of 3-Gy-irradiated GBM cells placed in SCM for long-term culture. Differentiated GBM cells treated or not by a 3-Gy irradiation and placed 2 days after in either FCS or SCM medium for long-term culture were subsequently orthotopically xenografted in nude mice to evaluate their tumorigenic potential. NS cells were also injected as a control for the stem condition, as they are enriched in GSC. (**a**) Survival curves established in xenografted mice for the indicated injected cell line (three mice per group for the D cell line and five mice per group for the G cell line). Exact *P-*values between the 3-Gy SCM group and the related CTR SCM group are indicated in the figure, after log-rank analysis. (**b**) Immunohistochemistry (IHC) analysis of Nanog-positive cell clusters in the brain tumors of killed, xenografted mice for the CTR SCM and the 3-Gy SCM groups (D and G cell lines, three mice per group). Shown are means±S.E.M. ^∗^*P*<0.05

One of the most important characteristics of GSC is their high tumorigenicity in orthotopically xenografted nude mice compared with non-stem cells.^43^ We analyzed accordingly the tumorigenic potential of long-term irradiated GBM cells cultured either in SCM or FCS medium at the end of the dedifferentiation protocol. We observed that GSC-enriched NS cells show the highest tumorigenic potential when compared with all differentiated conditions (Figure 6a). Three-Gray-irradiated cells kept in FCS medium failed to show a significant tumorigenicity increase compared with untreated cells. As control cells maintained in SCM medium showed a slight increase in their tumorigenic potential, we observed that only the 3-Gy-treated cells placed in SCM medium were able to display a markedly increased tumorigenicity, with survival curves relatively close to those of the corresponding NS cell line (Figure 6a). We next performed an immunolabeling of the stem factor Nanog in the brain of these xenografted mice and we showed that Nanog is expressed by small cell clusters, as already described for several stem markers in human tumor tissues,^44, 45^ and is significantly increased in the 3-Gy SCM group compared with the related control (Figure 6b and Supplementary Figure 2). Although these first elements need to be further confirmed in larger *in vivo* studies, they seem to support our observations establishing that IR could potentiate the dedifferentiation of GBM cells *in vitro* and lead to an upregulation of GSC number and an increased tumorigenicity *in vivo*.

### Requirement of a survivin-dependent pathway for IR-induced dedifferentiation in GBM cells

**Figure 7:**
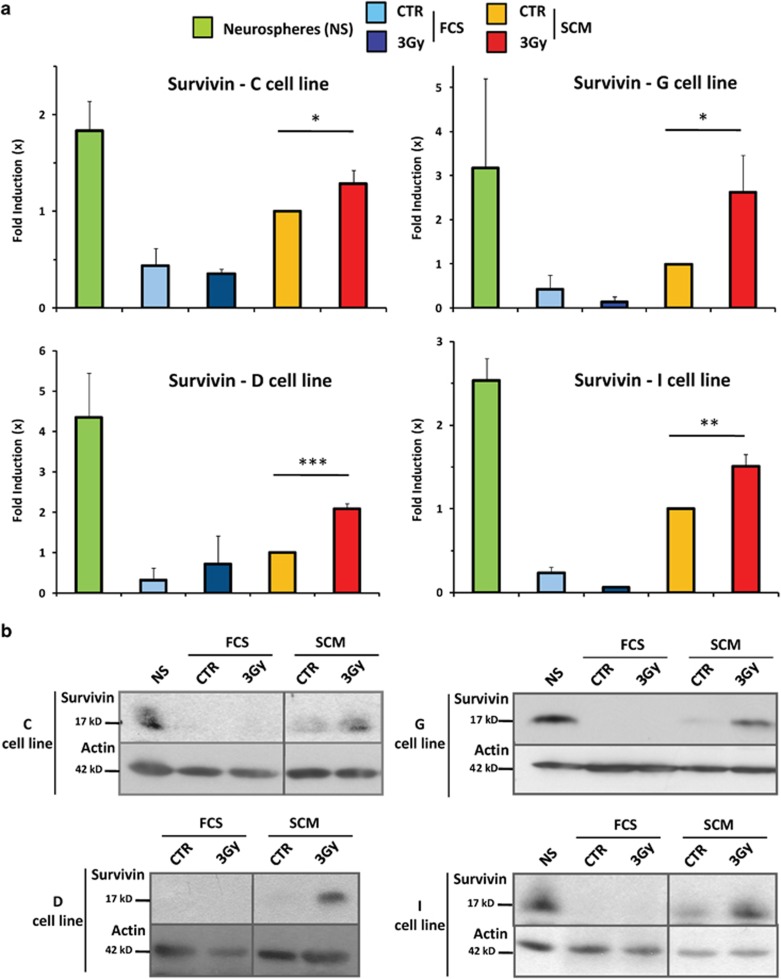
Overexpression of the anti-apoptotic protein survivin in 3-Gy-irradiated GBM-differentiated cells placed in SCM for long-term culture. Differentiated GBM cells treated or not by a 3-Gy irradiation and placed 2 days after in either FCS or SCM medium for long-term culture were analyzed for survivin expression either by real-time quantitative PCR (**a**) or western blotting (**b**) at the end of the dedifferentiation protocol and for the four different patient cell lines. RNA and protein expression levels for Survivin were also analyzed in NS cells as a control for the stem condition. (**a**) PCR results were expressed as fold inductions relative to the CTR SCM condition and shown as means±S.E.M. of at least three independent experiments. ^∗^*P*<0.05, ^∗∗^*P*<0.01, ^∗∗∗^*P*<0.001. (**b**) Western blotting results were representative of at least three independent experiments for each cell line. Equal gel loading and transfer efficiency were checked with an anti-actin antibody

Our group and others previously showed that survivin (BIRC5), an anti-apoptotic IAP, is involved in GBM cell radioresistance, notably in relation with hypoxia pathway.^32, 46^ Moreover, it appears that survivin has a major functional role in neural progenitor cells and during neurogenesis^47^ and is closely associated with the SonicHH/Gli1 pathway in GSC.^48^ In line with this, survivin was recently shown to be upregulated in GSC, to contribute to their cell death resistance and to be increased by IR.^49^ We then hypothesized that survivin may be involved in the IR-induced dedifferentiation process described above. We first checked the survivin expression in GSC-enriched NS, in differentiated cells and during the IR-induced dedifferentiation, by qPCR (Figure 7a) and western blotting (Figure 7b). We observed that in all the cell lines, this IAP was dramatically downregulated in FCS-differentiated cells compared with NS, independently of their irradiation status. Interestingly, a slight overexpression of survivin was seen in untreated SCM condition but only the 3-Gy condition allowed to overexpress survivin at a level approaching the one displayed in NS (Figures 7a and b). This increase was also observed at short term after irradiation (Supplementary Figure 1b). In consequence, these variations appeared to be very similar to those seen for the different stem markers analyzed above (Figures 4a and 5a, and Supplementary Figure 1a).

**Figure 8:**
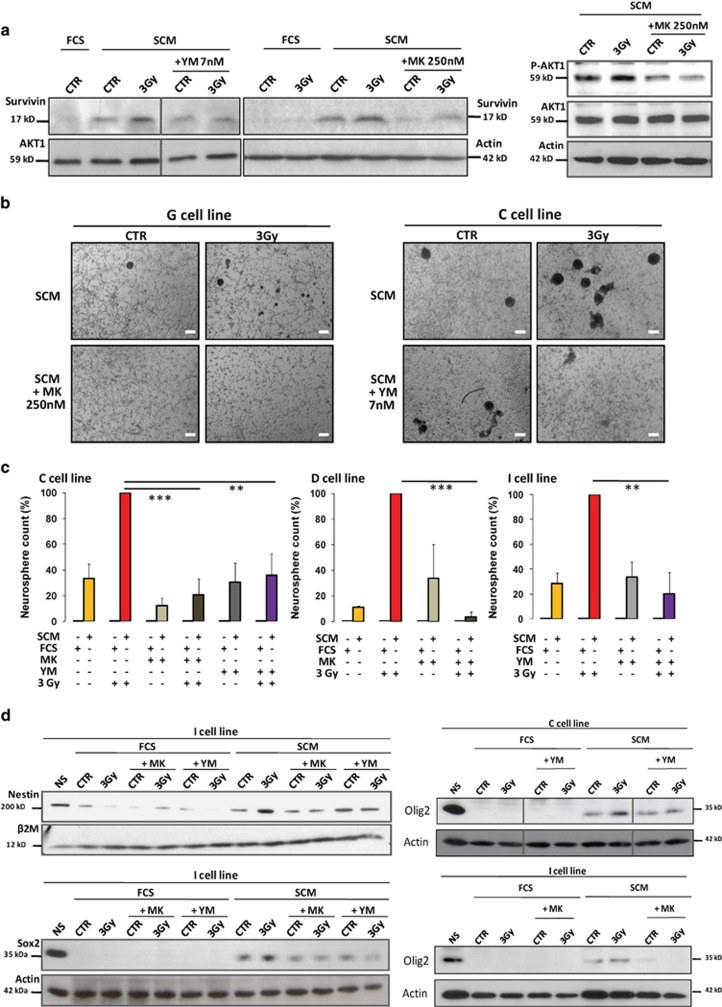
Requirement of the IR-induced survivin overexpression for the dedifferentiation process in GBM cells. (**a**–**d**) Differentiated GBM cells were pre-treated for 24 h with either a survivin inhibitor (YM-155 7 nM) or an AKT inhibitor (MK-2206 250 nM) and then irradiated or not at 3 Gy before being placed 2 days after in either FCS or SCM medium, complemented or not with fresh YM-155 or MK-2206 inhibitors. (**a**) Western blot analysis of the effect of YM-155 and MK-2206 treatment on Survivin expression in GBM cells (I cell line) irradiated or not and kept in SCM medium for 1 additional week. Efficiency of MK-2206 toward AKT was also checked as a control by the blotting of phospho-AKT1 (pAKT1). (**b** and **c**) At the end of the dedifferentiation protocol, the effects of YM-155 and MK-2206 were measured on the NS generation potential in response to IR by NS counting in phase-contrast microscopy (original magnification: × 4, scale bar: 17 *μ*m) (**b**) and subsequent quantification in the indicated cell lines (**c**). Results are expressed as the means±S.E.M. of three independent experiments. ^∗∗^*P*<0.01, ^∗∗∗^*P*<0.001 compared with the 3-Gy SCM condition. (**d**) The involvement of Survivin in the IR-induced GBM reprogramming was checked by western blotting by analyzing the expression of the stem markers Nestin, Sox2 and Olig2 at the end of the dedifferentiation protocol in the presence or absence of YM-155 and MK-2206. Concerning western blottings, equal gel loading and transfer efficiency were checked with an anti-actin, AKT1 or *β*2-microglobulin (*β*2M) antibody, and results were representative of at least three independent experiments on the indicated cell line and reproduced in all the cell lines

We then investigated whether this survivin overexpression during the IR-induced dedifferentiation was a consequence of this reprogramming or could be an essential step supporting IR-induced GBM plasticity to a stem-like phenotype. To this end, we treated GBM cells during the dedifferentiation process with 7 nM YM-155, a selective inhibitor of survivin used in anti-cancer clinical trials.^50^ As an additional control, we used MK-2206 (250 nM), a selective AKT inhibitor,^51^ as we and others^52^ showed that AKT can control the expression of survivin in GBM cells (Figure 8a). We first checked the efficiency of these inhibitors used at non-toxic concentrations (data not shown) on survivin expression by western blotting during the IR-promoted dedifferentiation (Figure 8a). Next, we observed their effect at the end of the dedifferentiation protocol and showed that both inhibitors induced a potent blockade of the 3-Gy-induced NS generation in SCM medium (Figures 8b and c). Moreover, YM-155 and MK-2206 markedly inhibited at the protein level the overexpression of the stem markers Olig2, Sox2 and Nestin in response to IR in SCM medium (Figure 8d). Altogether, these results strongly suggest that the IR-induced reprogramming in GBM cells was associated and supported by the upregulation of the anti-apoptotic protein survivin.

## Discussion

Radiotherapy is undoubtedly a key component for GBM treatment. The standard protocol defined by Stupp *et al.*^53, 54^ associates concomitant chemotherapy with Temozolomide and radiotherapy at a total dose of 60 Gy (30 daily fractions of 2 Gy), followed by adjuvant TMZ chemotherapy. This combined chemo/radiotherapy following surgical tumor resection, when possible, leads to a median overall survival of 14.6 months in the EORTC-NCIC trial^53, 54^ and the relapse, almost inevitable, mainly occurs at the initial tumor location exposed to radiotherapy.^55^ Consequently, it appeared essential to identify the possible origins of this high relapse rate and to increase the efficacy of radiotherapy in GBM to improve their clinical prognosis.

Growing number of studies deciphered the resistance pathways occurring in GBM cells in response to IR and notably focused on the radioresistant GSC subpopulation, much more resistant to IR compared with differentiated GBM cells.^6, 13, 16^ These GSC then may be selected by the treatment to favor the subsequent tumor regrowth and relapse. Different targeted strategies have been already tested to radiosensitize GSC in order to suppress either their survival ability or their tumorigenic competency.^6, 12, 13, 16^ Forced differentiation was also used to sensitize GSC to radiotherapy.^56, 57^ Nevertheless, none of these radiosensitizing strategies got interested to target a putative dedifferentiation mechanism occurring in resistant differentiated cells remaining in the tumor site after combined surgery/radiochemotherapy and to analyze the effects of IR with regard to the cellular plasticity processes.

For the first time, we demonstrated here that IR at a subtoxic 3-Gy dose close to the daily used dose in clinic are responsible for favoring at long term a major dedifferentiation process in GBM cells, with the gain of an *in vitro* self-renewal ability, the overexpression of a large panel of stem markers, the dramatic decrease of several well-established neural differentiation markers and finally the acquisition of a potentiated tumorigenic potential *in vivo* in orthotopically xenografted nude mice. Moreover, we showed that this cellular plasticity to a cancer stem cell (CSC) phenotype occurred independently of any clonal selection, as we did not observe any impairment of either the proliferation rate or the cell viability after a 3-Gy irradiation along the whole dedifferentiation process. Altogether, our data highlight the existence of a new mechanism of radioresistance in GBM cells through a cellular adaptation of the surviving cancer cells after treatment, leading to their reprogramming to a stem-like state much more tumorigenic. This work supports some recent observations in breast cancer cells showing the re-acquisition of several stem characters in response to IR, highlighting in some ways their reprogramming toward a stem-like phenotype.^58^

This new process of cell adaptation to radiotherapy, which allows remaining differentiated cancer cells to acquire stemness, may probably contribute, together with clonal selection, to the stem cell compartment expansion inside the tumor after treatment^41^ and to the fast and almost inevitable recurrence of these tumors. In consequence, it could be of great interest to specifically inhibit this particular IR-induced plasticity in order to setup new clinical strategies, in the aim to optimize radiotherapy in GBM patients. For this purpose, we analyzed several genes known to participate to a cellular adaptation process in response to IR and notably the anti-apoptotic protein survivin, recently highlighted by our group in irradiated GBM cells.^32^ We showed in the present study that survivin was markedly enhanced in GSC compared with their differentiated counterparts, and that its expression was potentiated during the dedifferentiation process in a similar way to the stem markers. Moreover, we and others previously showed that this IAP is involved in GBM radioresistance,^32, 46^ is increased by IR in GBM cells,^46, 49^ is upregulated in GSC compared with differentiated GBM cells,^49^ is associated with faster GBM recurrence^49^ and is overexpressed in GBM recurrence tumor samples compared with newly diagnosed ones.^49^ In addition, survivin was also shown to be tightly associated with different stem-promoting pathways in CSC/NSC, notably SonicHH/Gli1,^48^ Notch,^59^ Oct4/Stat3^60^ and Sox2.^61^ In consequence, we investigated its role in the IR-induced GBM cell dedifferentiation. Through the use of the selective inhibitor of survivin YM-155, actually used in phase II clinical trials for advanced non-small cell lung carcinoma, melanoma, breast and prostate cancer,^62^ we established that survivin is essential to the occurrence of the IR-induced plasticity process and sustains both the NS-forming ability and the stem markers overexpression in response to IR. It would be of interest to further study the precise role of this IAP in the dedifferentiation process, as it appears that survivin has, in addition to its inhibitory role of the executioner caspases during apoptosis,^63^ several other roles in the cell machinery such as the mitosis process^63^ or the DNA-damage repair system.^64^ Finally, survivin was also shown to be a major target of different pro-survival signaling pathways such as the PI3K/AKT axis and the HIF factors.^65^ As AKT and HIFs were shown to sustain the stem-like GSC phenotype,^23, 24, 66, 67^ this warrants future studies to discover the interplay between IR, hypoxic signaling and AKT/survivin to install the GBM dedifferentiation process.

Altogether, our data demonstrate that a clinically relevant radiation dose, a key component of the conventional GBM treatment, can potentiate in differentiated GBM cells the acquisition of a stem-like phenotype associated with increased *in vitro* self-renewal capacity and *in vivo* tumorigenesis. This plasticity process in response to IR appeared to be supported by survivin, known to be tightly associated with several stem-maintaining pathways. This survivin-mediated reprogramming to stemness could probably contribute to the expansion of the GSC compartment after treatment and may favor the fast recurrence of these aggressive brain tumors. Setting up new clinical strategies to restrain this IR-induced dedifferentiation should be considered for forthcoming trials, and on this basis the specific targeting of survivin may be an interesting approach.

## Materials and methods

### Human tumor collection

The study was conducted on newly diagnosed GBM tumor samples isolated from four different patients to establish four primary GSC cell lines (C, D, G and I). For FACS sorting experiments, a fifth GSC patient cell line was also used (A). These samples were all obtained after written informed consent from patients admitted to the Neurosurgery Department at Toulouse University Hospital and were processed in accordance with the Institution's Human Research Ethics Committee. Tumors used in this study were histologically diagnosed as grade IV astrocytoma according to the WHO criteria. All the results depicted in this study were obtained from at least three different independent experiments in the same cell line and were reproduced in all the other cell lines.

### Cell culture

The GBM samples were processed as described by Avril *et al.*,^68^ in order to obtain the corresponding primary NS cell lines shown by other groups to be enriched in GSC.^29^ NS GSC lines were maintained in DMEM-F12 (Lonza, Levallois-Perret, France) supplemented with B27 and N2 (Invitrogen, Life Technologies, Saint Aubin, France), 25 ng/ml of FGF-2 and EGF (Peprotech, Neuilly sur Seine, France) at 37 °C in 5% CO_2_ humidified incubators. All GSC lines were used for the experiments in this SCM medium between the second and twelfth passages, in order to avoid any stem cell characteristic loss. Forced differentiation was performed according to previous published protocol^69^ adapted as follows. Briefly, the dissociated NS cells were cultured and plated as adherent monolayer (7.5 × 10^3^ cells/cm^2^) in DMEM-F12 supplemented only with 10% FCS (FCS medium) on laminin (1.5 *μ*g/cm^2^, Sigma-Aldrich, Saint-Quentin Fallavier, France) for at least 15 days to ensure an optimum differentiation.

### Irradiation-induced dedifferentiation protocol and NS generation assays

The differentiated cells were subjected or not to 3 (subtoxic dose) or 12 Gy (positive control for cell death and proliferation assays) (Gamma-cell Exactor 40, Nordion, Ottawa, ON, Canada). Two days post IR, cells were placed either in FCS or SCM medium to keep them differentiated or to favor the dedifferentiation process in a permissive stem medium, respectively (Figure 2a). Unless stated, cells were cultured until the appearance of a sufficient number of NS in the medium, corresponding to an average of 30 days after irradiation. For the experiments using the selective chemical inhibitors of survivin or AKT, 7 nM YM-155 (SelleckChem, Houston, TX, USA) or 250 nM MK-2206 (SelleckChem) were respectively added as a pretreatment 2 days before irradiation and then renewed with the cell medium every week. At the end of the protocol, cultures were observed by microscopy (Nikon Diaphot, Nikon, Champigny sur Marne, France) under a × 4 or × 10 objective and the primary NS were counted for each condition in the entire 25-cm^2^ flask (Figures 3a and b). Next, the totality of the cells was collected after trypsinization for subsequent experiments. For adaptation of the limiting dilution NS generation assay, we plated differentiated cells in 24-cell plates at different low cell densities (2500–30 000 cells/well) before subjecting them to the long-term protocol. Generated primary NS were then counted by microscopy in each well (Figure 3c). When stated, differentiated cells could also be sorted by FACS analysis to select the A2B5-negative cell population before running the primary NS generation assay (Figure 3d).

Primary NS obtained at the end of the dedifferentiation protocol, with or without a preliminary FACS sorting of the differentiated cells, were dissociated and plated in 96-well plates (24 wells per condition) at different cellular densities (1–500 cells/well), in order to assess their ability to generate secondary NS through limiting dilution assays. After 15 days, secondary NS were counted by microscopy in each well.

### Quantitative real-time RT-PCR

Total RNA was isolated either from primary NS, FCS-differentiated cells or from cells at the end of the dedifferentiation protocol using RNeasy kit (Qiagen, Courtaboeuf, France) and then reverse-transcribed using iScript cDNA synthesis kit (Bio-Rad, Marnes la Coquette, France). Real-time qPCR reactions were carried out using the Fluidigm 96.96 dynamic array integrated fluidic circuits and the Biomark HD System (Fluidigm, Les Ulis, France) according Advanced Development Protocol no. 37 (Toulouse GeT Platform). *β*2-Microglobulin was used as endogenous control in the ΔCt analysis. The different primers (Eurogentec, Angers, France) used in this study are described in Supplementary Table 1.

### Immunohistochemistry

Immunohistochemistry was performed on the excised brains on paraffin-embedded sections (5 *μ*m). For Nanog detection, only the brain samples displaying an equivalent tumor area of 70–80% of the total brain were selected, as determined by Hemalun–Eosin staining (Supplementary Figure 2a). Briefly, the sections were incubated for 90 min with an anti-Nanog antibody (Ab62734, Abcam, Paris, France). Slides were counterstained with hematoxylin and viewed on a Nikon microscope. Nanog-positive cell clusters were then counted in the tumor area.

### Flow cytometry analyses

Direct immunofluorescence assay was performed by FACS as previously described.^70, 71^ When required, collected cells were first subjected to a step of permeabilization using the cytofix/cytoperm kit (BD Biosciences, Le Pont de Claix, France). For all samples, 2 × 10^5^ cells were then incubated for 30 min in PBS with 10% BSA at 4 °C to avoid nonspecific binding, and then incubated with appropriate conjugated primary antibodies for 40 min at 4 °C. Fluorescence related to immunolabeling was measured using a FACSCalibur flow cytometer (BD Biosciences). The antibodies used are depicted in Supplementary Table 2. Each measurement was conducted on at least 7000 events, acquired on CellQuest software (BD Biosciences) and analyzed with VenturiOne software (Applied Cytometry, Sheffield, UK). To evaluate the marker expression, we determined the specific fluorescence index (SFI) using the mean fluorescence intensity (MFI). The SFI was calculated as previously described, with the following formula SFI=(MFI antibody−MFI isotype control)/MFI isotype control.^71^ The gating strategy used in these analyses is described in Supplementary Figure 3 and is based on a previously published protocol.^72^ For NS generation assay, a FACS sorting (Beckman MoFlo Astrios, Beckman Coulter, Villepinte, France) was performed when mentioned on GBM-differentiated cells (A cell line) before the assay, in order to only sort the differentiated population, which was characterized by (i) its specific FSC-H/SSC-H pattern (Gate C, see Supplementary Figure 3) and (ii) its negative expression of the stem marker A2B5, as previously described.^4, 73^

### Western blotting

Cells were lysed in RIPA buffer complemented with cocktails of protease and phosphatase inhibitors (Sigma-Aldrich). Twenty-five micrograms of proteins were then separated on a 10 or 12.5% SDS-PAGE, electroblotted onto PVDF membranes (Amersham, GE Healthcare, Velizy-Villacoublay, France), which were blocked with 10% milk. The primary antibodies used for this study are listed in Supplementary Table 3.

### Cell death and proliferation assays

Apoptotic cells were quantified using FACS analysis by determining the percentage of cells with subG1-DNA content. This subG1 population was analysed after cell permeabilization and subsequent PI staining, as previously described.^74^ Apoptosis and necrosis were also quantified at the same time on non-permeabilized cells by flow cytometry, with an Alexa Fluor 488-conjugated AV and PI kit, according to the manufacturer's protocol (Invitrogen, Life Technologies). SubG1 and AV measurements were conducted on at least 10 000 events, acquired on CellQuest software (BD Biosciences) and analyzed with VenturiOne software (Applied Cytometry). The proliferation rate was finally analyzed using the WST-1 assay (Roche Diagnostics, Meylan, France) in 96-well microplates, as previously described.^70^ The yellow formazan product formed by viable adherent cells was quantified by detection of its absorbance at 450 nm using a Multiskan Multisoft Labsystem spectrophotometer (Thermofisher, Illkirch, France). For these cell death and proliferation assays, differentiated cells were treated or not with a 3- or 12-Gy irradiation, placed 2 days after either in FCS or SCM medium and collected for analysis 1 week after irradiation.

### Orthotopic xenograft generation

Nude mice were housed in the Claudius Regaud Institute Animal Care-accredited facility and the Institution animal ethics committee approval was obtained for the use of the animal model and the study protocols. Orthotopic human GBM xenografts were established in 4- to 6-week-old female nude mice (Janvier Labs, Le Genest-Saint-Isle, France) as previously described.^68^ Briefly, mice received a stereotaxically guided injection of either 1.75 × 10^5^ (G cell line) or 2.5 × 10^5^ cells (D cell line) collected at the end of the dedifferentiation protocol and resuspended in DMEM-F12. The injection was precisely located into the right forebrain (2 mm lateral and 1 mm anterior to the bregma at a 5-mm depth from the skull surface). In order to check the cell tumorigenicity, survival curves were established and mice were killed at the appearance of neurological signs. Excised brains were then collected for subsequent immunohistochemistry analysis.

### Statistical analysis

The results are presented as means±S.E.M. of at least three independent experiments. Significant differences (^∗^*P*<0.05, ^∗∗^*P*<0.01 and ^∗∗∗^*P*<0.001) were evaluated with the Student *t*-test. Log-rank analysis of Kaplan–Meier survival curves was used to evaluate the tumorigenesis of injected cells, with *P*<0.05 considered as significantly different (Graphpad Prism v5, GraphPad Software, La Jolla, CA, USA). Western blottings and FACS plots are representative of at least three different experiments in the same cell line and were reproduced in all the other cell lines.

## Abbreviations

CSC: cancer stem cell
GBM: glioblastoma
GFAP: glial fibrillary acidic protein
GSC: GBM stem-like cell
HIF: hypoxia-inducible factor
IAP: inhibitor of apoptosis protein
IR: ionizing radiation
NS: neurosphere
NSC: neural stem cell
SCM: stem cell medium
SCTF: stem cell transcription factor
SonicHH: Sonic hedgehog
