# Supplementary material for: Ionizing radiations sustain glioblastoma cell dedifferentiation to a stem-like phenotype through survivin: possible involvement in radioresistance
Source: Cell Death Dis. 2014 Nov 27;5(11):e1543–. doi: 10.1038/cddis.2014.509 (PMC4260760; doi:10.1038/cddis.2014.509)
Supplement: Supplementary Figures [file cddis2014509x1.pdf]

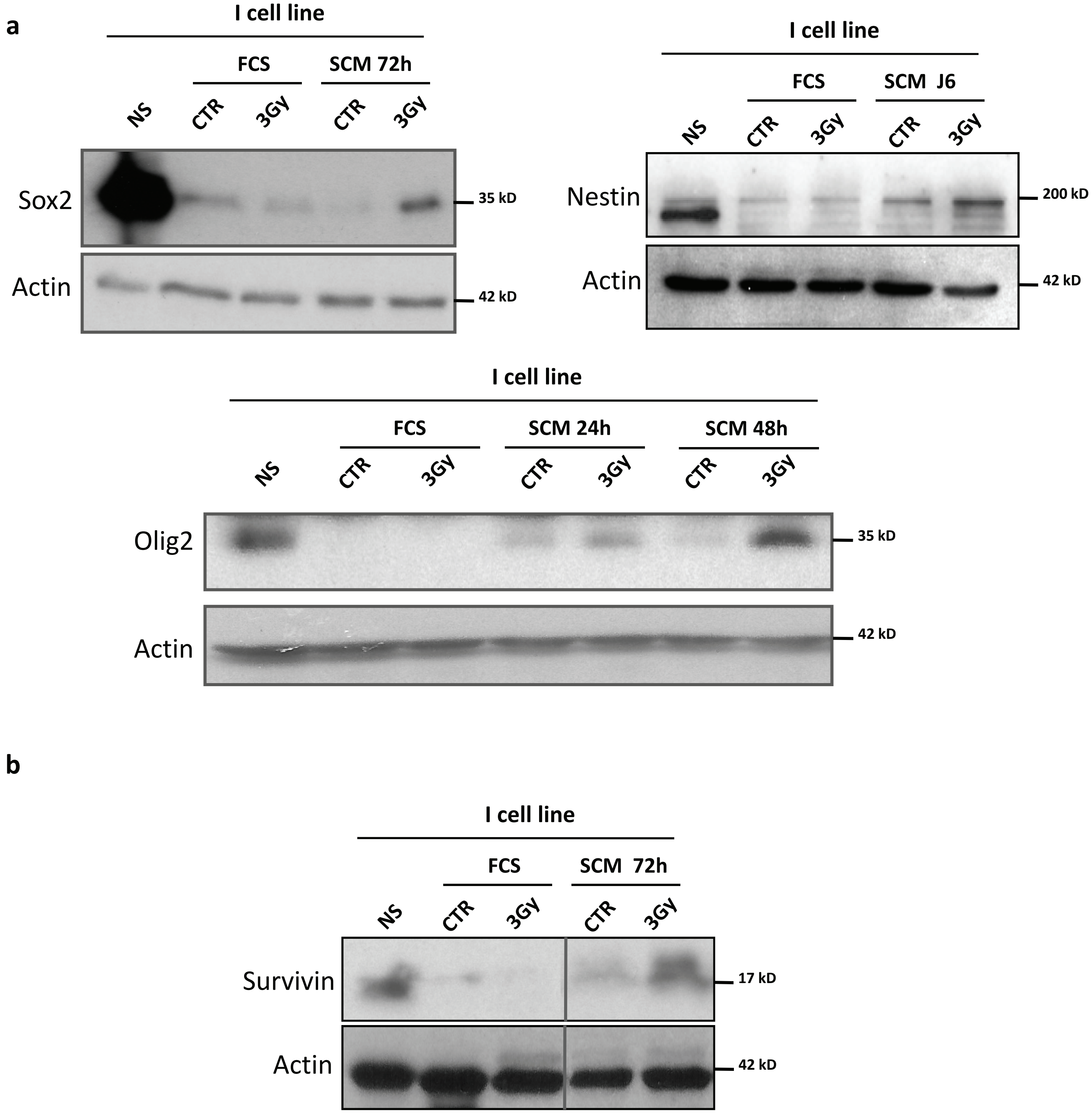

Supplementary Figure 1: Overexpression of stem markers Sox2, Olig2 and Nestin (a) and survivin (b) at short term in GBM cells after a 3 Gy-irradiation. Differentiated GBM cells treated or not by a 3 Gy-irradiation and placed 24h after in either FCS or SCM medium for 1 to 6 supplemental days were analyzed by Western-blot. Protein expression levels in neurosphere cells were shown as a control for the stem condition. Equal gel loading and transfer efficiency were checked with anti-actin.

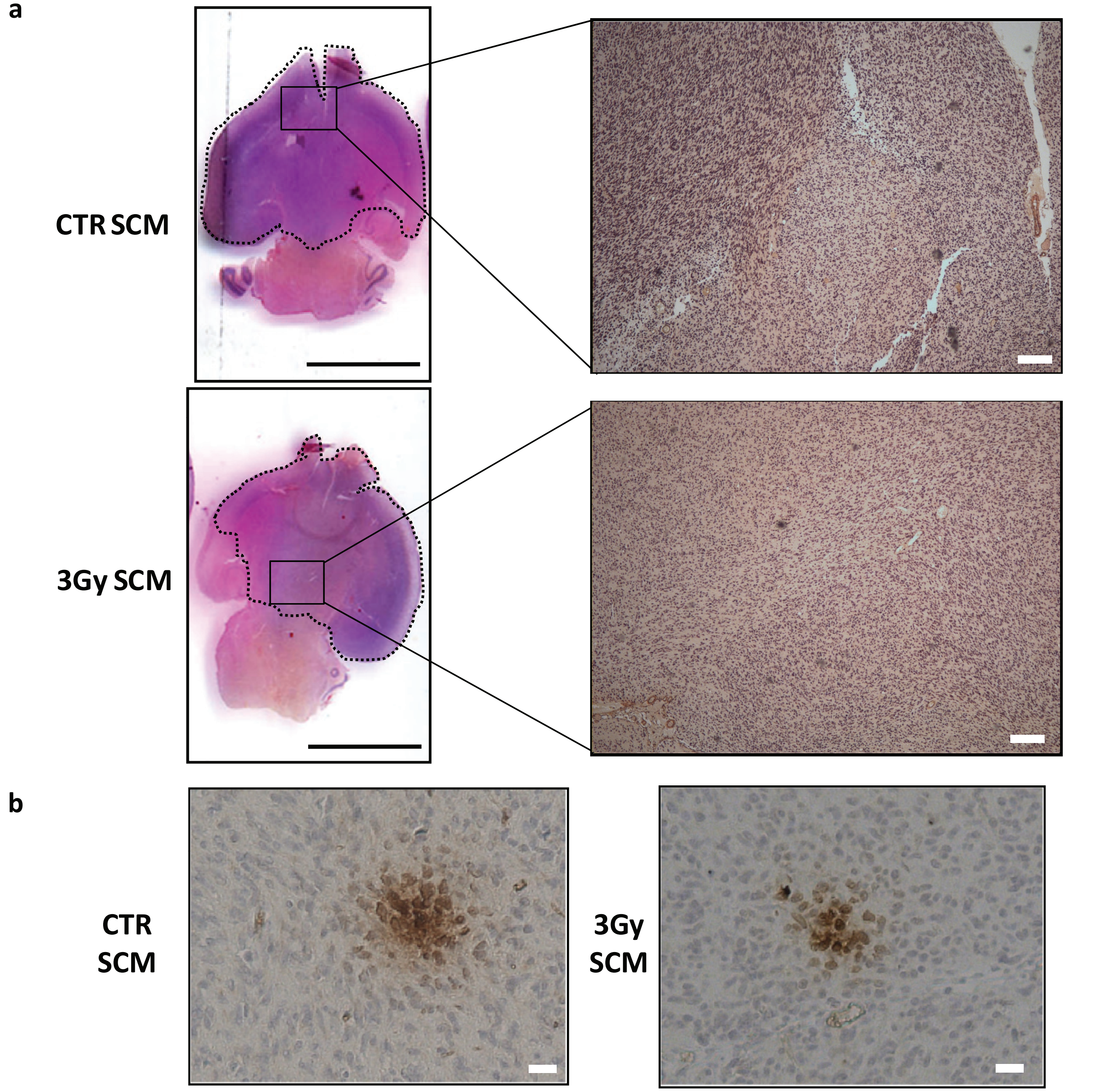

Supplementary Figure 2: Hemalun-Eosin staining of orthotopically xenografted GBM tumor cells in nude mice brain. Differentiated GBM cells (G cell line) treated or not with a 3Gy-irradiation and placed 2 days after in SCM medium for long-term culture were subsequently orthotopically xenografted in nude mice to evaluate their tumorigenic potential. At the appearance of neurological signs, mice were sacrificed and their brain were collected to perform paraffin-embedded sections. **(a)** Prior to IHC, a Hemalun-Eosin staining was done to estimate the area of the tumor (delineated borders) and only the samples comprised between 70-80% of the total brain were selected for IHC in order to compare the different conditions. A representative staining photomicrograph was shown for the whole brain section (right panel, scale bar : 0.5 cm) and a HE-positive tumoral area (right panel, magnification: x4, scale bar: 12.5 µm). **(b)** Nanog expression pattern by IHC in orthotopically xenografted tumors obtained after injection of GBM cells (G cell line) subjected to the dedifferentiation process in SCM with or without a 3Gy irradiation. A representative IHC photomicrograph was shown for each condition. Magnification: x10, scale bar: 5 µm.

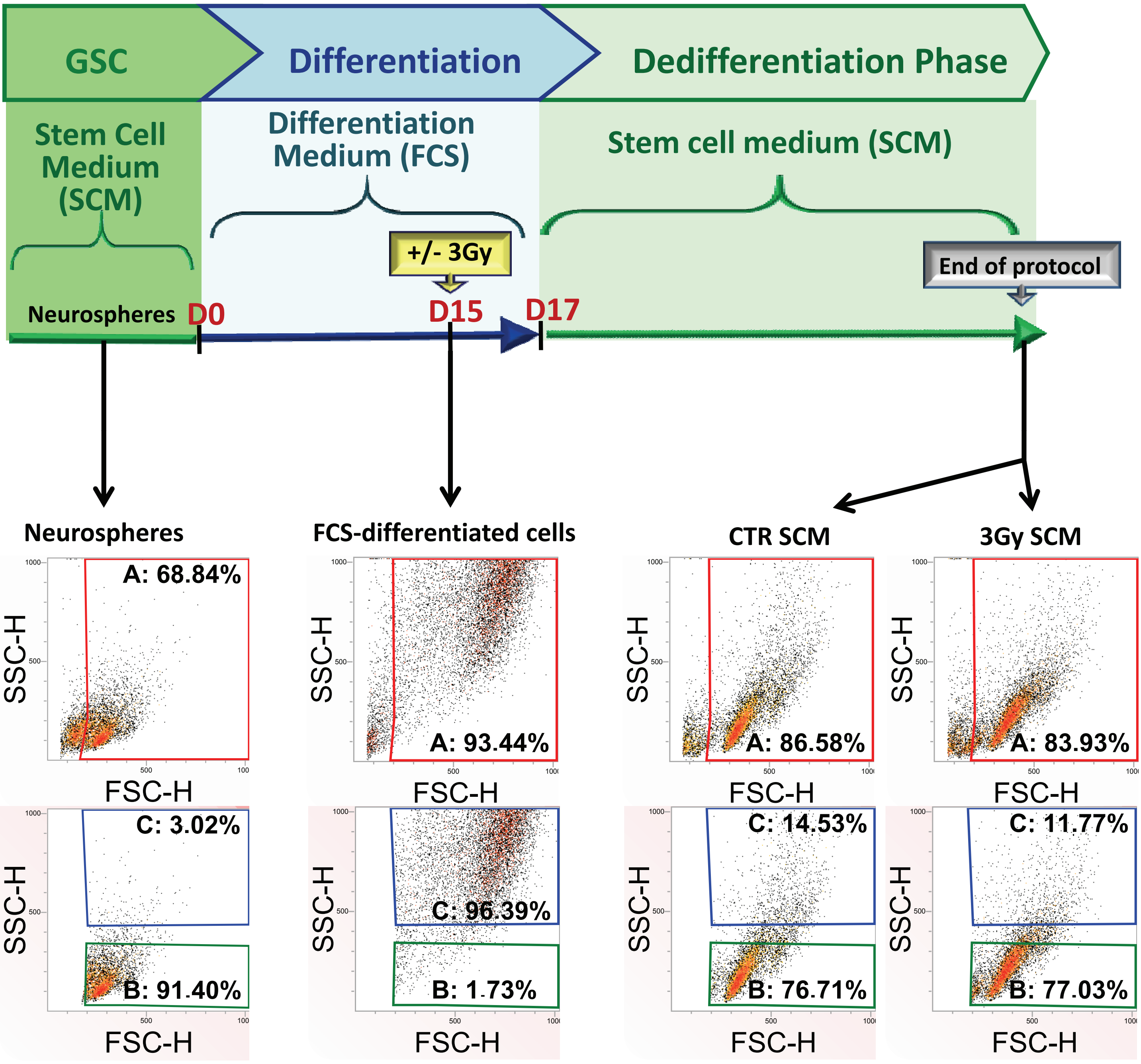

Supplementary Figure 3: Gating strategy used for the direct immunofluorescence assay by FACS. GBM cells were gated and then analyzed by FACS according to the protocol previously described (Mlynarik et al., 2012) in order to discriminate the less-differentiated population (Gate B) from the well-differentiated population (Gate C). Prior to this, a pre-gating was performed to avoid cell debris in the analysis (Gate A). In the neurosphere cells used for this study, the percentage of cells in Gate B was always comprised between 70 to 100%, as they are enriched in GSC. The 15 days-differentiation process in FCS medium led to a well-differentiated cell population superior to 90% (Gate C). At the end of the dedifferentiation process in SCM medium, the harvested cells irradiated or not were mostly located in Gate B (70 to 100%). As a consequence, immunofluorescence analyses were performed on cells located in Gate B for Neurospheres and SCM-dedifferentiated cells and on Gate C for the FCS-differentiated cells.
