# Supplementary material for: Ionizing radiations sustain glioblastoma cell dedifferentiation to a stem-like phenotype through survivin: possible involvement in radioresistance
Source: Cell Death Dis. 2014 Nov 27;5(11):e1543–. doi: 10.1038/cddis.2014.509 (PMC4260760; doi:10.1038/cddis.2014.509)
Supplement: Supplementary Tables [file cddis2014509x2.doc]

**Supplementary tables**

**Supplementary Table** 1. Forward and reverse primer sequences used for quantitative PCR detection.

|  | **Quantitative PCR primer sequences** |
| --- | --- |
| **β2-microglobulin (human)** | forward 5’-acccccactgaaaaagatga-3’  reverse 5’- atcttcaaacctccatgatg-3’ |
| **Cd133 (human)** | forward 5’-GCATTGGCATCTTCTATGGTT-3’  reverse 5’- CGCCTTGTCCTTGGTAGTGT-3’ |
| **CTGF (human)** | forward 5’-CATCTCCACCCGGGTTACC-3’  reverse 5’-CAGGCGGCTCTGCTTCTCTA-3’ |
| **EZH2 (human)** | forward 5’-GGACCACAGTGTTACCAGCAT-3’  reverse 5’-GTGGGGTCTTTATCCGCTCAG-3’ |
| **GFAP (human)** | forward 5’-GGCAAAAGCACCAAAGACGG-3’  reverse 5’-GGCGGCGTTCCATTTACAAT-3’ |
| **Gli1 (human)** | forward 5’-AATGCTGCCATGGATGCTAGA-3’  reverse 5’-GAGTATCAGTAGGTGGGAAGTCCATAT-3’ |
| **Nanog (human)** | forward 5’-GTCCCGGTCAAGAAACAGAA-3’  reverse 5’-TGCGTCACACCATTGCTATT-3’ |
| **Nestin (human)** | forward 5’-ATCGCTCAGGTCCTGGAAGG-3’  reverse 5’-AAGCTGAGGGAAGTCTTGGAG-3’ |
| **Notch1 (human)** | forward 5’-TGGACCAGATTGGGGAGTTC-3’  reverse 5’-GCACACTCGTCTGTGTTGAC-3’ |
| **Olig1 (human)** | forward 5’-AGGTAACCAGGCGTCTCACAGT-3’  reverse 5’-CGGTACTCCTGCGTGTTAATGA-3’ |
| **Olig2 (human)** | forward 5’-CAGAAGCGCTGATGGTCATA-3’  reverse 5’-TCGGCAGTTTTGGGTTATTC-3’ |
| **OMG (human)** | forward 5’-TAGGGACTCCATGTTCTACCCA-3’  reverse 5’- TCTGCATCCCACTTACAGTGA-3’ |
| **SonicHH (human)** | forward 5’-GCGGAAGGTATGAAGGGAAG-3’  reverse 5’-GCCAAAGCGTTCAACTTGTC-3’ |
| **Sox2 (human)** | forward 5’-GCACATGAACGGCTGGAGCAACG-3’  reverse 5’-TGCTGCGAGTAGGACATGCTGTAGG-3’ |
| **Survivin (human)** | forward 5’-CGAGGCTGGCTTCATCCA-3’  reverse 5’-AGAAGAAACACTGGGCCAAGTC-3’ |

**Supplementary Table** 2. Primary antibodies used for flow cytometry.

| **Marker** | **Antibodies and fluorochromes** | **Suppliers** | **FACS channel** |
| --- | --- | --- | --- |
| **A2B5 (human)** | Mouse monoclonal antibody : A2B5 - APC  Isotype control : IgM - APC | Miltenyi | FL4-H |
| **Cd133 (human)** | Mouse monoclonal antibody : Cd133/2 - PE  Isotype control : Igg2B -PE | Miltenyi | FL2-H |
| **GFAP (human)** | Mouse monoclonal antibody : GFAP - AF647  Isotype control : Igg2B - AF647 | BD Biosciences | FL4-H |
| **Nanog (human)** | Goat polyclonal antibody : Nanog - PE  Isotype control : IgG - PE | R&D Systems | FL2-H |
| **Nestin (human)** | Mouse monoclonal antibody : Nestin - Fluorescein  Isotype control : Igg1 - Fluorescein | R&D Systems | FL1-H |
| **Notch1 (human)** | Mouse monoclonal antibody : Notch1 - PerCP  Isotype control : Igg1 - PerCP | R&D Systems | FL3-H |
| **O4 (human)** | Mouse monoclonal antibody : O4 - PE  Isotype control : IgM - PE | R&D Systems | FL2-H |
| **TUJ1 (human)** | Mouse monoclonal antibody : Tuj1 - AF488  Isotype control : Igg2A - AF488 | BD Biosciences | FL1-H |
| **Sox2 (human)** | Mouse monoclonal antibody : Sox2 - APC  Isotype control : Igg2A - APC | R&D Systems | FL4-H |

**Supplementary Table 3. Primary antib**odies used for western blotting.

| **Marker** | **Antibodies** | **Suppliers** |
| --- | --- | --- |
| **Actin (human)** | Mouse monoclonal antibody (MAB1501) | Millipore |
| **β2-microglobulin (human)** | Rabbit monoclonal antibody (Ab75853) | Abcam |
| **AKT1 (human)** | Rabbit monoclonal antibody (Ab32505) | Abcam |
| **AKT1 (phospho S473) (human)** | Rabbit polyclonal antibody (Ab66138) | Abcam |
| **Nestin (human)** | Mouse monoclonal antibody (MAB5326) | Millipore |
| **Olig2 (human)** | Rabbit polyclonal antibody (AB9610) | Millipore |
| **Sox2 (human)** | Mouse monoclonal antibody (MAB4343) | Millipore |
| **Survivin (human)** | Rabbit monoclonal antibody (Ab76424) | Abcam |
